# Lesion Profiling at Primary Isolation in RIII Mice is Insufficient in Distinguishing BSE from Classical Scrapie

**DOI:** 10.1111/j.1750-3639.2009.00273.x

**Published:** 2010-03

**Authors:** Katy E Beck, Melanie Chaplin, Michael Stack, Rosemary E Sallis, Sarah Simonini, Richard Lockey, John Spiropoulos

**Affiliations:** Veterinary Laboratories Agency—WeybridgeNew Haw, Addlestone, Surrey, KT15 3NB, UK

**Keywords:** BSE, mouse bioassay, prion, RIII mice, scrapie

## Abstract

Primary isolation of bovine spongiform encephalopathy (BSE) in RIII mice generates a lesion profile believed to be reproducible and distinct from that produced by classical scrapie. This profile, which is characterized by peaks at gray matter areas 1, 4 and 7 (dorsal medulla, hypothalamus and septal nuclei), is used to diagnose BSE on primary isolation. The aim of this study was to investigate whether the BSE agent could be present in sheep diagnosed with classical scrapie, using lesion profiles in RIII mice as a discriminatory method. Sixty-two positive scrapie field cases were collected from individual farms between 1996 and 1999 and bioassayed in RIII mice. Fifty-five of these isolates transmitted successfully to at least one mouse. Of the 31 that produced adequate data to allow lesion profile analysis, 10 showed a consistent profile with peaks at brain areas 1, 4 and 7. All inocula for this subgroup were derived from sheep of genotype ARQ/ARQ. While the 1-4-7-scrapie profile exhibited similarities to BSE in RIII mice at primary isolation, it was distinguishable based on histopathology, immunohistochemistry and cluster analysis. We conclude that caution should be taken to distinguish this profile from BSE and that additional parameters should be considered to reach a final diagnosis.

## INTRODUCTION

Scrapie and bovine spongiform encephalopathy (BSE) are examples of transmissible spongiform encephalopathies (TSEs), also known as prion diseases. They are fatal, neurodegenerative diseases characterized by vacuolar degeneration in the central nervous system (CNS), deposition of an abnormal conformer of host encoded prion protein, PrP^Sc^, amyloid plaque formation, neuronal loss and gliosis. While classical scrapie has long been endemic in the UK national flock, it is not believed to represent a public health risk. Emergence of BSE as a scrapie-like disease in cattle was, however, relatively recent (26). BSE presented a huge challenge to the veterinary community, confounded by evidence for a link between BSE and new variant Creutzfeldt-Jakob Disease (vCJD) (6, 9, 21), which affects humans following exposure to the BSE agent (6). The feeding of cattle with foodstuffs contaminated with scrapie remains one of the main hypotheses for the emergence of BSE in Great Britain (27, 28).

The mouse bioassay represents the gold standard method for investigation of the biologic properties of prions and the discrimination of TSE strains. Primary passage of ovine isolates to mice typically results in low attack rates and longer incubation periods because of the species barrier. Following subpassage and stabilization, individual mouse-adapted TSE strain types can be distinguished based on reproducible incubation periods and patterns of vacuolation unique to specific lines of mice following inoculation via a consistent route. Furthermore, quantitation of vacuolation in specific neuroanatomic areas of infected mouse brains produces lesion profiles that permit comparison of vacuolation patterns between TSE isolates following passage to mice (18). Based on the bioassay method using wild type inbred mouse lines, BSE was shown to be caused by a single strain of agent that produced a unique lesion profile and incubation period (17).

Previous studies based on limited sample sizes suggested that consistent lesion profiles do not arise from scrapie isolates on primary isolation (5, 6) and that subpassage is considered necessary for strain stabilization and full strain characterization. In contrast, the BSE agent produces a unique and highly reproducible lesion profile when transmitted to RIII mice on primary isolation, characterized by peaks in vacuolation score in the dorsal medulla, the hypothalamus and the septum. More significantly, it has been reported that, under similar conditions, scrapie generates lesion profiles that are highly variable from each other and distinct from the BSE profile (6). The stability of the BSE lesion profile in RIII mice on primary isolation (6) and the subsequent identification of two stable strains, 301C and 301V, depending on which of the two PrP genotypes of mouse is used on subpassage (8), has been attributed to the existence of a single major strain of the agent in the original host. In contrast, there are several different mouse-adapted scrapie strains indicating the presence of more than one classical scrapie strain in the original host (7). One possible explanation for the different behaviors of these two TSE agents may be that the BSE agent has emerged recently and is able to cross species barriers without any alteration of its biologic properties. In contrast, scrapie is highly adapted to sheep, and different strains of the agent may have evolved over time and adapted to several host-specific factors, the most important being the PrP genotype. The BSE lesion profile in RIII mice is independent of the source of the BSE agent (direct transmission from bovine- or ovine- or porcine-passaged BSE), inoculation of CNS or non-CNS material, inocula prepared from experimental or natural cases, inocula derived from pooled material or individual brains (20), or the titer of the inoculum (19). Lesion profiles from RIII mice challenged with vCJD are indistinguishable from those caused by BSE but distinct from those induced by sporadic CJD, a form of CJD that preexisted the BSE outbreak (6). Also, the ability to reproduce this profile from cases of spongiform encephalopathy in captive ruminants and from cases of feline spongiform encephalopathy played a crucial role in establishing BSE as the causative agent of these syndromes (5). Subsequently, this characteristic profile has contributed significantly to the confirmation of BSE from a natural TSE case in a goat (13). The RIII mouse line has therefore been adopted by the World Organization for Animal Health (OIE) and the Community Reference Laboratory as one of the ultimate means to discriminate BSE from scrapie in ovine samples with an ambiguous diagnosis (29).

The association of vCJD with BSE and the successful experimental transmission of BSE to sheep raised the possibility that BSE may have entered the national flock. Although the characteristics of experimental transmission of BSE to sheep have been studied fully, it is not known with certainty if the experimental model would adequately reflect natural BSE infection in sheep. To investigate if the BSE agent could be present in sheep diagnosed with scrapie, a significant number of ovine samples that were confirmed as classical scrapie with histopathology, immunohistochemistry and Western blot were subjected to bioassay in RIII mice, and the lesion profiles were used as a discriminatory method. Here we report the results of these transmissions and discuss their scientific and policy implications.

## MATERIALS AND METHODS

### Animal selection

Clinical scrapie suspects from commercial farms throughout England and Wales were transported live to a regional Veterinary Laboratories Agency laboratory between 1996 and 1999. After euthanasia, the brain was removed aseptically, the medulla at the level of the obex was fixed for statutory diagnosis and the remaining brain was frozen for biochemical analysis and bioassay studies. Each case was also blood-sampled for PrP genotyping at codons 136, 154 and 171. Clinical cases that were confirmed positive for classical scrapie by statutory tests [histopathology, immunohistochemistry (IHC) and Western blot] were included in the project as they were reported. Each farm contributed either a single or two cases of different PrP genotype in an effort to include as many different farms as possible and avoid overrepresentation of farms with a high incidence of scrapie. With the exception of a single case, ARQ/VRQ (A = Alanine, R = Arginine, Q = Glutamine, V = Valine) animals were required to be removed from this study to produce a brain pool required by another project. No other selection criteria were applied.

### Western immunoblotting

Fresh brain tissue from the 31 scrapie inocula and five ovine BSE samples were processed, electrophoresed and transferred by using the BioRad TeSeE Western blot kit (BioRad, Marnes-la-Coquette, France) according to the manufacturer's instructions. Samples were probed by using either monoclonal antibody (mAb) SHA31 or mAb P4, and the signal was detected by using a Fluor-S multiImager (BioRad). Glycoform profiling was carried out by using Quantity One analysis software (BioRad).

### Mouse inoculation and sample preparation

A 10% (w/v) medulla homogenate prepared in sterile saline was inoculated into RIII mice, 20 µL via intracerebral and 100 µL via intraperitoneal routes. All work was carried out in accordance with the Animals (Scientific Procedures) Act 1986. Twenty mice aged 6 to 10 weeks were used per inoculum. Mice were monitored from 250 days post inoculation for clinical signs of TSE infection, which included weight loss, incontinence, affected gait, vacant stare and a rough coat. Mice were euthanized by using carbon dioxide when clinical end–point had been reached, defined as having received positive clinical scores of TSE in 2 consecutive weeks or having received scores of “definitely affected” in 2 out of 3 consecutive weeks. Mice were also euthanized if there was significant deterioration, lack of mobility or inability to eat or drink at any time during the monitoring process. Brains were removed and fixed in 10% neutral buffered formalin for at least 3 days at room temperature before being cut into five coronal levels to reveal caudal medulla, rostral medulla, midbrain, thalamic and frontal levels, required for lesion quantitation and profiling as detailed below. Tissues were processed and embedded in paraffin wax by using routine histological methods. Sections (3 µm) were subsequently mounted on the same slide for interpretation.

### Histopathological and immunohistochemical analysis

Sections were stained with hematoxylin and eosin (H&E) as previously detailed (20). Post-mortem TSE diagnosis was confirmed based on the presence of characteristic neuropil vacuolation. Lesion severity in TSE positive samples was further quantitated by using an established method (18). In brief, scores were assigned on a scale of 0–5, for specific neuroanatomic gray matter areas of the brain that were then plotted to produce lesion profiles. Vacuolation was scored based on its intensity in each area as detailed in other published work(20). Areas scored were G1, dorsal medulla nuclei; G2, cerebellar cortex of the folia including the granular layer, adjacent to the fourth ventricle; G3, cortex of the superior colliculus; G4, hypothalamus; G5, thalamus; G6, hippocampus; G7, septal nuclei of the paraterminal body; G8, cerebral cortex (at the level of G4 and G5); and G9, cerebral cortex (at the level of G7). Because of variation in individual mouse profiles, the mean profile for each inoculum was calculated if it included five or more mice with positive clinical scores. For IHC, samples were labeled with rabbit polyclonal antibody Rb486 that recognizes amino acids 221–233 of the bovine prion protein using a standard method (10).

### Statistical analysis of lesion profile and incubation period data

Average lesion profiles produced from different TSE isolates were compared by using cluster analysis (Statistica 7.0 statistical software, StatSoft Inc., Tulsa, OK, USA). Results were visualized as a dendrogram. Comparison of mean incubation period between groups of inocula was performed by using two-tailed *t*-tests, where significance was accepted at *P* < 0.01 or *P*< 0.05.

## RESULTS

### Western immunoblotting

**Figure 1:**
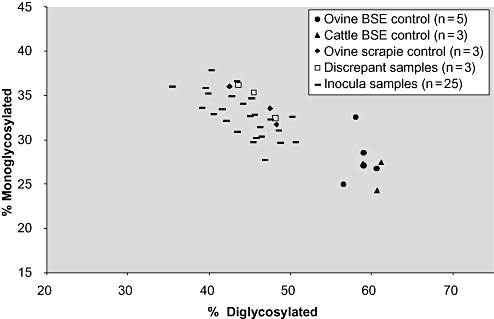
Glycoform ratio chart showing the ovine and bovine BSE samples with a higher percentage of diglycosylated PrP^Sc^ compared with the ovine scrapie controls and inocula. Abbreviation: BSE = bovine spongiform encephalopathy.

Three criteria are used in the differential analysis of the PrP^Sc^ molecular profiles: (i) the molecular mass of the three characteristic protein bands obtained for PrP^Sc^; (ii) differential immunoreactivity ratio between the signals obtained with an antibody raised to the core region of the abnormal protein (mAb Sha31) and an antibody raised to the N-terminal region (mAb P4); and (iii) by measuring the glycoform ratio of the signals obtained for the di- and monoglycosylated protein bands. For BSE, samples show a lower molecular mass for the unglycosylated band and strong reactivity with a core antibody and little or no reactivity with an N-terminal antibody. Scrapie samples, however, exhibit a higher molecular mass and strong reactivity with both core and N-terminal antibodies. Glycoform ratio analysis tends to show ovine and bovine BSE giving the higher percentage of di-glycosylated PrP^Sc^ than that of ovine scrapie(24). The intensity and quality of the PrP^Sc^ molecular profile were suitable for full analysis in 28 of the 31 cases tested. The remaining three cases were unsuitable for glycoform analysis (parameter iii) but were classified as classical scrapie based on parameters i and ii. Using this combination of parameters, some variability was seen regarding a lower molecular migration of the unglycosylated band of PrP^Sc^ in six samples with 0.9kD to 2.1kD lower measurements of the unglycosylated band than those obtained for the scrapie controls (all six were ARQ/ARQ genotype), and three of these also exhibited a more marked reduction of the mAb P4 signal than the scrapie controls (results not shown). However, the glycoform ratios for all 28 samples were grouped with the scrapie controls in the scattergram and were distinct from the bovine BSE and BSE in sheep control samples (Figure 1). As none of these six samples fulfilled all of the criteria found in sheep experimentally infected with BSE by Western immunoblotting (24, 25), they were all classified as scrapie.

### Lesion profiles

**Figure 2:**
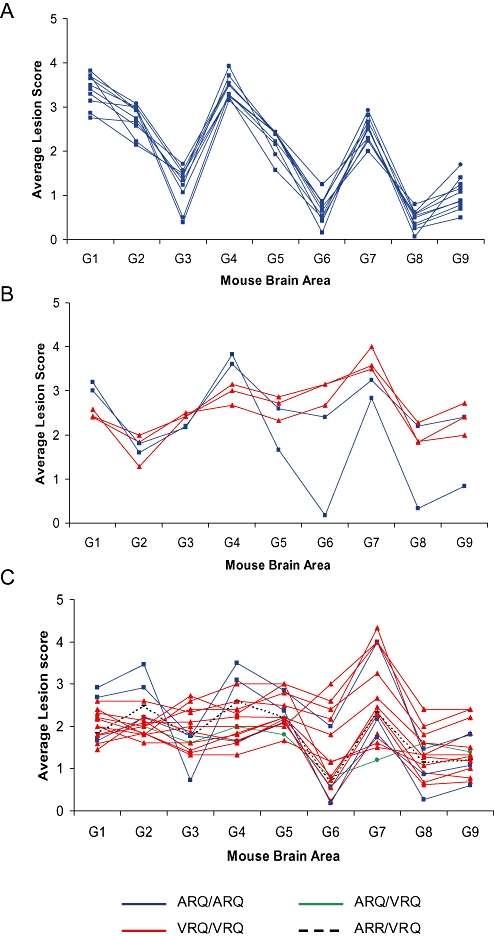
*Average lesion profiles in RIII mice of 31 inocula with ≥5 mice per inoculum.***A.** 10 scrapie sources, all derived from ARQ/ARQ sheep, gave a 1-4-7 profile (1-4-7-scrapie). **B.** 5 scrapie sources gave alternative 1-4-7 profiles (1-4-7-subgroup 2). **C.** 16 scrapie sources gave non-1-4-7 profiles (non-1-4-7-subgroup). Profiles were obtained following quantitation of specific vacuolation in nine neuroanatomic gray-matter areas: G1, dorsal medulla nuclei; G2, cerebellar cortex of the folia including the granular layer, adjacent to the fourth ventricle; G3, cortex of the superior colliculus; G4, hypothalamus; G5, thalamus; G6, hippocampus; G7, septal nuclei of the paraterminal body; G8, cerebral cortex (at the level of G4 and G5); and G9, cerebral cortex (at the level of G7).

Of the 62 positive classical scrapie field cases that were used to inoculate mice, seven did not transmit to RIII mice. Of the remaining 55 isolates, 24 transmitted to less than five clinically and H&E positive mice per inoculum and were not included for further study as lesion profiles constructed from less than five mice are not reliable. Thirty-one inocula transmitted to give five or more positive mice with analyzable lesion profiles (Table 1). Table 2 shows the results of the remaining primary transmissions to RIII mice, which were not included in the current analysis. Average lesion profiles for each of the 31 inocula were initially plotted and are shown in Figure 2. Ten inocula showed a consistent profile with peaks at brain areas 1, 4 and 7 (termed 1-4-7-scrapie). All inocula for this group were derived from sheep of PrP genotype ARQ/ARQ (Figure 2A). Using the 1-4-7 profile as a selection criterion, the remaining 21 isolates presented as two further groups according to lesion profile. A group of five inocula gave alternative 1-4-7-profiles (termed 1-4-7-subgroup 2) and was derived from ARQ or VRQ homozygotes (Figure 2B). While peaks were again evident at brain areas 1, 4 and 7, lesion scoring in brain area 3 was higher than that in area 2, in contrast to 1-4-7-scrapie where the opposite was observed. A third non-1-4-7 profile was associated with the V allele in 13 of 16 inocula (termed non-1-4-7-subgroup) (Figure 2C).

**Table 2:** Results of scrapie transmissions from isolates with less than five clinically and H&E-positive mice suitable for lesion profile analysis. Abbreviations: BSE = bovine spongiform encephalopathy; H&E = hematoxylin and eosin.

Source code	Ovine genotype	Number (incubation period range in days)	
		Clinically + ve, pathologically + ve	Clinically − ve, pathologically + ve
Scrapie 22*	VRQ/VRQ	8 (405–569)	2 (473–504)
Scrapie 39*	VRQ/VRQ	5 (421–718)	8 (502–776)
Scrapie 11	VRQ/VRQ	4 (572–674)	4 (516–593)
Scrapie 27	VRQ/VRQ	4 (565–749)	0 (–)
Scrapie 35	VRQ/VRQ	4 (402–700)	2 (853–871)
Scrapie 87	VRQ/VRQ	3 (388–764)	1 (610)
Scrapie 16	VRQ/VRQ	2 (553–639)	1 (622)
Scrapie 20	ARQ/VRQ	2 (685–721)	3 (502–722)
Scrapie 38	VRQ/VRQ	2 (435–490)	2 (431–776)
Scrapie 51	ARQ/ARQ	2 (482–665)	5 (406–761)
Scrapie 76	VRQ/VRQ	2 (490–639)	4 (567–645)
Scrapie 79	VRQ/VRQ	2 (527–647)	10 (527–753)
Scrapie 2	AHQ/ARQ	1 (474)	2 (861–964)
Scrapie 6	ARQ/ARQ	1 (653)	1 (776)
Scrapie 7	ARQ/VRQ	1 (454)	0 (–)
Scrapie 15	ARQ/VRQ	1 (677)	0 (–)
Scrapie 18	VRQ/VRQ	1 (465)	0 (–)
Scrapie 70	ARQ/ARQ	1 (676)	0 (–)
Scrapie 3	ARQ/VRQ	0 (–)	1 (844)
Scrapie 4	VRQ/VRQ	0 (–)	1 (677)
Scrapie 16	VRQ/VRQ	0 (–)	1 (622)
Scrapie 68	VRQ/VRQ	0 (–)	1 (432)
Scrapie 74	ARQ/ARQ	0 (–)	1 (620)
Scrapie 78	ARQ/ARH	0 (–)	1 (847)
Scrapie 88	ARQ/ARQ	0 (–)	1 (774)
Scrapie 1	ARQ/ARQ	0 (–)	0 (–)
Scrapie 21	ARQ/VRQ	0 (–)	0 (–)
Scrapie 23	ARQ/ARQ	0 (–)	0 (–)
Scrapie 29	ARQ/ARQ	0 (–)	0 (–)
Scrapie 71	VRQ/VRQ	0 (–)	0 (–)
Scrapie 85	ARQ/ARQ	0 (–)	0 (–)

Each isolate was inoculated into 20 mice.

* Although these isolates had five or more clinically and pathologically positive mice, there were not enough coronal sections suitable for lesion profiling.

**Table 1:** Results of BSE and scrapie transmissions from isolates with five or more clinically and H&E-positive mice. Abbreviations: BSE = bovine spongiform encephalopathy; H&E = hematoxylin and eosin.

Source code	Ovine genotype	Number (incubation period range in days)	
		Clinically + ve, pathologically + ve	Clinically − ve, pathologically + ve
Scrapie 55	ARQ/ARQ	16 (419–476)	4 (397–454)
Scrapie 31	ARQ/ARQ	15 (429–503)	2 (377–460)
Scrapie 42	ARQ/ARQ	15 (424–491)	1 (431)
Scrapie 53	ARQ/ARQ	15 (415–442)	2 (376–382)
Scrapie 57	VRQ/VRQ	14 (484–610)	2 (479–764)
Scrapie 63	ARQ/ARQ	14 (459–533)	1 (477)
Scrapie 67	ARQ/ARQ	14 (421–549)	1 (546)
Scrapie 72	ARQ/ARQ	14 (443–516)	2 (450–486)
Scrapie 89	ARQ/ARQ	14 (375–443)	3 (366–430)
Scrapie 8	VRQ/VRQ	13 (448–566)	0 (–)
Scrapie 50	VRQ/VRQ	13 (459–542)	4 (350–461)
Scrapie 80	ARQ/ARQ	13 (308–413)	7 (280–408)
Scrapie 82	ARQ/ARQ	12 (428–503)	8 (380–451)
Scrapie 90	ARQ/ARQ	12 (394–458)	6 (280–475)
Scrapie 69	ARQ/ARQ	11 (419–462)	8 (436–459)
Scrapie 77	VRQ/VRQ	11 (396–521)	4 (433–511)
Scrapie 12	ARR/VRQ	10 (447–553)	5 (389–552)
Scrapie 73	VRQ/VRQ	10 (440–540)	4 (451–523)
Scrapie 19	ARQ/ARQ	9 (413–455)	11 (375–455)
Scrapie 49	VRQ/VRQ	8 (536–692)	5 (621–746)
Scrapie 84	VRQ/VRQ	7 (513–736)	3 (553–722)
Scrapie 14	ARQ/ARQ	6 (467–598)	7 (379–533)
Scrapie 43	VRQ/VRQ	6 (487–679)	7 (519–718)
Scrapie 48	VRQ/VRQ	6 (518–573)	4 (573–756)
Scrapie 58	VRQ/VRQ	6 (505–833)	6 (586–697)
Scrapie 59	ARQ/ARQ	6 (479–564)	11 (462–531)
Scrapie 83	VRQ/VRQ	6 (464–673)	4 (485–743)
Scrapie 5	ARQ/VRQ	5 (465–987)	4 (835–993)
Scrapie 75	VRQ/VRQ	5 (399–699)	6 (526–767)
Scrapie 81	VRQ/VRQ	5 (508–675)	6 (471–651)
Scrapie 86	VRQ/VRQ	5 (410–659)	5 (625–725)
Bovine BSE 97		17 (352–604)	2 (518–659)
Ovine BSE 14	ARQ/ARQ	17 (299–492)	0 (–)
Ovine BSE 15	ARQ/ARQ	16 (295–460)	0 (–)
Bovine BSE 98		16 (328–552)	0 (–)
Ovine BSE 31	ARQ/ARQ	15 (315–416)	0 (–)
Ovine BSE 32	ARQ/ARQ	15 (293–426)	3 (320–398)
Ovine BSE 111	ARQ/ARQ	14 (335–513)	2 (326–437)
Bovine BSE 96		14 (324–499)	2 (362–444)
Bovine BSE 186		12 (349–437)	5 (331–394)
Ovine BSE 30	ARQ/ARQ	11 (307–368)	0 (–)

Each isolate was inoculated into 20 mice.

**Figure 3:**
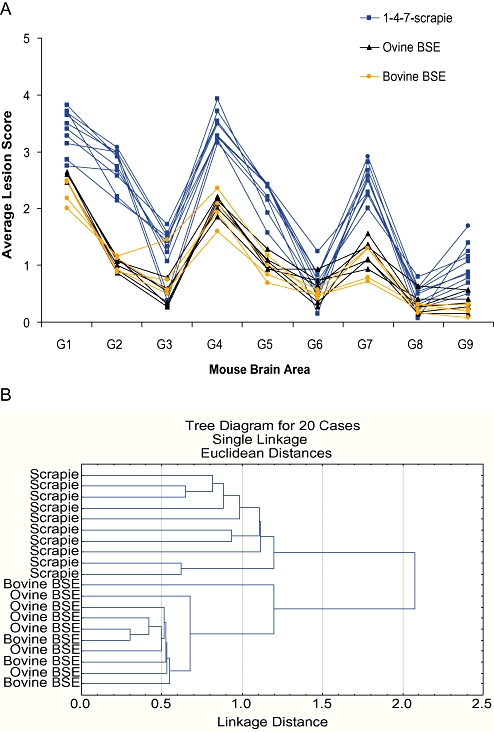
*The 1-4-7-scrapie profile is not consistent with BSE.***A.** Average lesion profiles for 1-4-7-scrapie isolates plotted against 10 BSE isolates (four bovine, six ovine passaged) for comparison. **B.** Tree diagram after cluster analysis (Statistica 7.0 statistical software, StatSoft Inc.) of the vacuolation scores in selected neuroanatomic brain areas to show that scrapie and BSE form two distinct groups as measured by Euclidean distance. Abbreviation: BSE = bovine spongiform encephalopathy.

**Figure 4:**
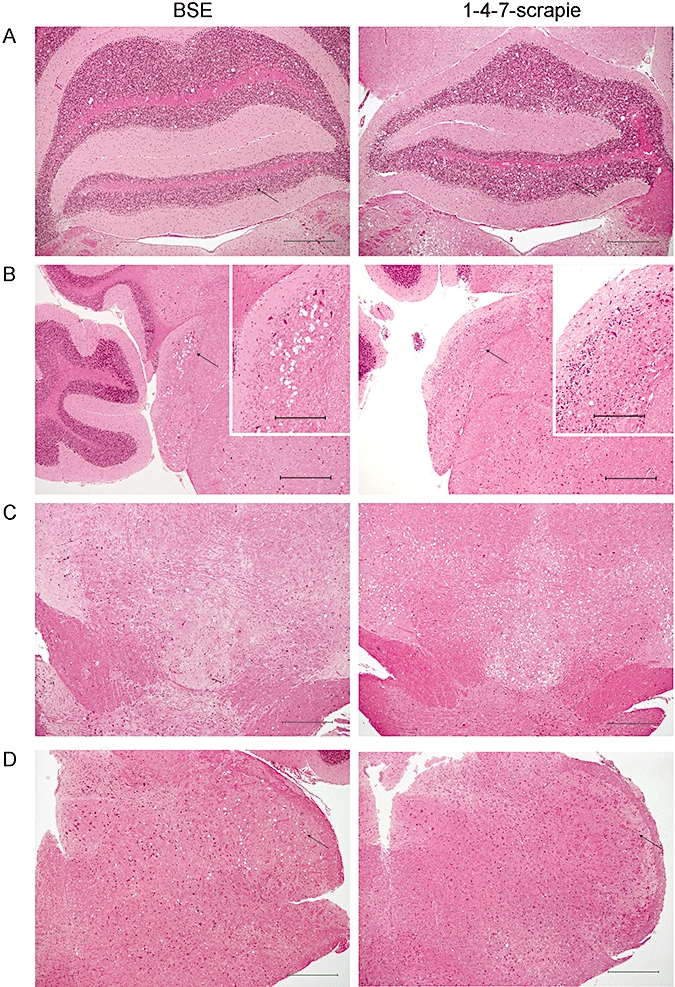
*Assessment of disease specific vacuolation by H&E staining revealed differences between BSE and 1-4-7-scrapie pathology.* Representative photographs are shown of **(A)** vacuolation of the granular layer of the cerebellum adjacent to the fourth ventricle (indicated by arrows), **(B)** the cochlear nuclei (indicated by arrows and shown at higher magnification), **(C)** the ventral midbrain and **(D)** the trigeminal nucleus (indicated by arrows). Scales bars represent 500 µm (main photographs) and 200 µm (inset photographs). Abbreviation: BSE = bovine spongiform encephalopathy.

The consistent peaks observed for 1-4-7-scrapie showed similarities to BSE previously profiled in RIII mice. In order to exclude unequivocally the possibility that the isolated agent was BSE, we analyzed these isolates further. In the first instance, for comparative purposes, the average profiles of the 10 identified 1-4-7-scrapie isolates were plotted with the average profiles of 10 BSE (six ovine-passaged BSE, four bovine BSE) isolates following primary isolation in RIII mice (Figure 3A). Both groups produced highly consistent lesion profiles. While similarities were apparent, the 1-4-7-scrapie profile was different to that of BSE in RIII mice. Scoring was consistently higher for the scrapie isolates, indicative of a more severe lesion: scores in brain areas 1 and 4 are >3 in scrapie isolates, whereas all brain areas score below 3 in BSE samples. Notably, vacuolation of brain area 2 (cerebellar cortex of the folia including the granular layer, adjacent to the fourth ventricle) was a characteristic of 1-4-7-scrapie that was not consistent with BSE: This area scored 2.7 ± 0.12 (mean score of the 10 isolates ± SEM) for 1-4-7-scrapie, consistent with vacuoles convincingly diagnostic for TSE. In contrast, BSE isolates gave a mean score of 1.0 ± 0.04 in area 2, signifying the observation of a few vacuoles that, in isolation, do not convincingly confirm TSE pathology. This was supported by histopathological findings (Figure 4). To confirm the distinction between 1-4-7-scrapie and BSE, lesion profiles (Figure 3A) were compared by using cluster analysis. Results showed 1-4-7-scrapie and BSE clustered as two distinct groups (Figure 3B).

### Pathology reveals differences between 1-4-7-scrapie sources and BSE

Having established that 1-4-7-scrapie was not consistent with BSE by cluster analysis despite similarities in lesion profile, we further analyzed the histopathology of 1-4-7-scrapie in comparison with BSE on primary isolation in RIII mice, also noting additional neuroanatomic areas/distinguishing features outside of the scoring areas. Five mice per inoculum were analyzed in detail, representing the highest, lowest, median, 25th and 75th percentile for incubation period. Analysis of H&E stained samples indicated that the overall lesion severity of 1-4-7-scrapie was more intense than that of BSE. It should be noted that bovine and ovine BSE were indistinguishable from each other, whereas there was a degree of heterogeneity between 1-4-7-scrapie samples, despite the presence of consistent features. Plaques were associated with all but one scrapie inoculum that gave a 1-4-7 profile, which subsequently showed plaques with IHC. They were located frequently in the corpus callosum or alveus, adjacent to the hippocampus. Plaques were absent from BSE isolates analyzed. The G2 scoring area was generally spared from vacuolation in BSE samples (Figure 4A). In contrast, G2 was generally vacuolated in 1-4-7-scrapie samples. The remaining granular layer of the cerebellum (excluding G2) was generally vacuolated in both 1-4-7-scrapie and BSE. Moderate/heavy vacuolation of the dorsal cochlear nuclei has been reported as a characteristic feature of BSE (20). This pattern of vacuolation was not observed in any of the 1-4-7-scrapie isolates (Figure 4B). Moderate/heavy vacuolation, with large and sometimes confluent, irregular-shaped vacuoles, was frequently observed in 1-4-7-scrapie in the ventral midbrain encompassing the interpeduncular nuclei, the substantia nigra and the white matter dorsal to these areas (Figure 4C). Conversely, while BSE isolates also exhibited vacuolation in the ventral midbrain, these vacuoles ranged in size and were generally evenly spread. Although not a consistent finding, the presence of large, rounded vacuoles in the trigeminal nucleus of the caudal medulla was indicative of BSE (Figure 4D). Within the thalamic section, the distribution of vacuolation was similar in 1-4-7-scrapie and BSE, but the lesion intensity was noticeably greater in 1-4-7-scrapie. The hippocampus was generally free of vacuolation in both BSE and scrapie isolates.

**Figure 5:**
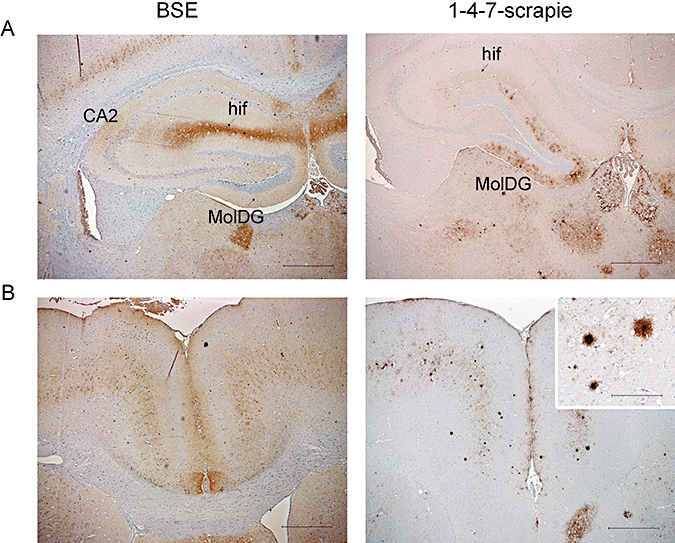
*Assessment of abnormal PrP deposition by immunohistochemistry confirmed histopathological findings.* Representative photographs are shown of **(A)** PrP^Sc^ deposition in the hippocampus, targeted to the molecular layer of the dentate gyrus (MolDG) for 1-4-7-scrapie but to the hippocampal fissure (hif) or at CA2/hif end for BSE isolates, **(B)** the cerebral cortex: plaques are a consistent feature of 1-4-7-scrapie but not BSE. Scale bars represent 500 µm (main photographs) and 100 µm (inset photographs). Abbreviation: BSE = bovine spongiform encephalopathy.

IHC analyzes supported histopathological differences between BSE and 1-4-7-scrapie. 1-4-7-scrapie was distinguishable from BSE by differential PrP^Sc^ deposition in the hippocampus: deposition was targeted to the molecular layer of the dentate gyrus (MolDG) in 1-4-7-scrapie isolates but generally appeared in the hippocampal fissure (hif) or at the CA2/hif end in BSE samples (Figure 5A). Plaques were a consistent feature of 1-4-7-scrapie, associated with all isolates and were frequently observed in the thalamus and the cerebral cortex. Plaques were not observed following primary isolation of BSE in RIII mice (Figure 5B).

### Incubation period analysis

**Figure 6:**
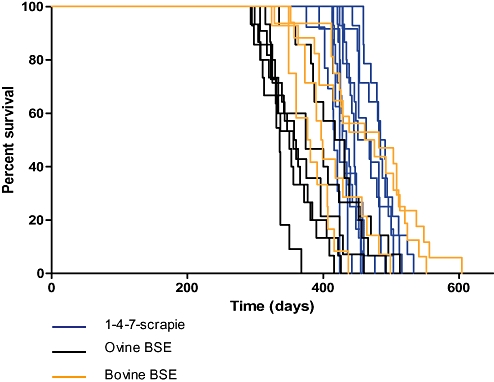
Survival curves of individual 1-4-7-scrapie and BSE sources (five or more clinically and H&E-positive mice per inoculum) are shown. Statistical comparison of the mean incubation period between groups was performed using the two-tailed *t*-test, where significance was accepted at *P* < 0.01 or *P* < 0.05. Abbreviations: BSE = bovine spongiform encephalopathy; H&E = hematoxylin and eosin.

To further resolve the difference between the 1-4-7-scrapie and BSE groups, mean incubation period was calculated for each subgroup of mice as follows [mean incubation period (days) ± SEM]: 1-4-7-scrapie, 449 ± 7.8; 1-4-7-subgroup 2, 566 ± 22.9; non-1-4-7-subgroup, 543 ± 19.8; ovine BSE, 369 ± 11.3; bovine BSE, 431 ± 13.9. The 1-4-7-scrapie isolates and the BSE isolates were plotted as survival curves (Figure 6). While the mean incubation period of the 1-4-7-scrapie group was significantly lower than that of 1-4-7-subgroup 2 and the non-1-4-7-subgroup, it remained significantly higher than the ovine BSE control group (*P* < 0.005, two-tailed *t*-test where significance was accepted at *P* < 0.01). Conversely, there was no significant difference between the mean incubation period of 1-4-7-scrapie and bovine BSE isolates. Mean incubation periods for ovine and bovine BSE differed significantly (*P* = 0.048, where significance was accepted at *P* < 0.05).

## DISCUSSION

The emergence of BSE in cattle and its ability to transmit under experimental conditions to sheep with clinical signs indistinguishable from scrapie mean that discrimination of scrapie from BSE through the mouse bioassay is important and forms an integral part of European TSE surveillance (14). The data presented in this study relate to 10 isolates diagnosed as classical scrapie that gave rise to a 1-4-7-lesion profile on primary isolation in RIII mice. Notably, all were derived from ARQ/ARQ sources. Two further groups of profiles were observed. Because these profiles were in no way indicative of BSE, no further analysis was required. The observation of consistent profiles is contrary to earlier publications that report scrapie profiles to be variable on primary isolation (6, 8). The difference between previous studies and the data presented could be a result, in part, of the lower sample sizes previously used and the prevalence of the VRQ allele in those samples (6, 8). The lesion profile of BSE in RIII mice is stable on primary isolation, is considered pathognomonic of the disease and has been extensively characterized following collation of 150 transmission experiments of BSE, or BSE-derived infections, to RIII mice (20). This profile has provided evidence for the existence of a single TSE strain within the BSE agent that can retain its properties upon experimental passage through other species (6, 20). To this end, RIII mice have been considered a particularly useful discriminatory tool for BSE with emphasis placed on this characteristic profile (29). This was recently shown in a study where the lesion profile produced in RIII mice was used in conjunction with Western blot analyses to identify the first BSE signature in a French goat (13).

The shape of the ARQ/ARQ scrapie profile in RIII mice observed in this study exhibited similarities with the characteristic profile of BSE on primary isolation, sharing peaks at gray-matter areas 1, 4 and 7. This profile has not previously been reported for classical scrapie. Histopathological analysis of 1-4-7-scrapie isolates revealed marked differences when compared with BSE. Vacuolation of G2, absence of dorsal cochlear nuclei vacuolation, heavy vacuolation of the ventral midbrain and the presence of plaques were particular features of 1-4-7-scrapie that distinguished these isolates from BSE. Immunohistochemical analysis confirmed the distinction between isolates: plaques were associated with all 1-4-7-scrapie isolates but not with BSE. The absence of plaque formation in RIII mice has been previously attributed to the BSE agent (4, 20). However, this finding is not applicable to all mouse lines. Florid plaques were identified as a characteristic of BSE infection in certain transgenic mouse lines (11). A difference in the PrP^Sc^ deposition pattern within the hippocampus was observed: PrP^Sc^ deposition was targeted to the molecular layer of the MolDG in 1-4-7-scrapie isolates but targeted to the hif and CA2 in BSE isolates in agreement with previous published work (4, 19).

All the isolates included in the current study, which, on primary transmission in RIII mice, gave rise to the 1-4-7-scrapie profile, were sourced from ARQ/ARQ sheep diagnosed with classical scrapie. However, two ARQ/ARQ isolates gave “alternative” 1-4-7-subgroup 2 profiles, while a further three gave non-1-4-7 profiles. These data suggest that disease phenotype in mice during primary isolation can be determined by factors other than the combination of the PrP genotype of the donor, at least at the three codons which are usually determined, and the host. These factors may include properties of the agent strain, polymorphisms in the host PrP sequence other than at codons 136, 154 and 171, the rest of the host genome as well as further parameters yet to be defined (7, 22). An early study on primary transmission of classical scrapie in RIII mice failed to generate consistent lesion profiles, and, furthermore, none of the resultant profiles bore any resemblance to BSE (6). However, only one of the 10 classical scrapie isolates included in that study derived from an ARQ/ARQ sheep, and this failed to transmit to wild-type mice (8). We believe that the detection of the 1-4-7-profile from classical scrapie cases in our study is a result of the large number of samples we analyzed representing the most commonly affected PrP genotypes.

Four isolates within 1-4-7-subgroup 2 showed similar profiles, while one isolate exhibited a distinct profile, which shared characteristics with 1-4-7-scrapie and BSE. We assigned this case into subgroup 2 (Figure 2B) because G3 scored higher than G2 in contrast to the isolates presented in Figure 2A. Cluster analysis of all isolates showing profiles with peaks at areas 1, 4 and 7 revealed that this isolate is more akin to 1-4-7-scrapie (unpublished data). This discrepancy may be because, during visual analysis, we used specific brain areas to separate the isolates, while cluster analysis considers the entire profile. Interestingly, in a single bovine BSE case, G3 was higher than G2 (Figure 3A). Despite the similarities between these two profiles, IHC analysis showed that the aberrant scrapie isolate was consistent with 1-4-7-scrapie.

Despite the large number of sheep included in this study, we could not collect enough data for robust statistical analysis from certain genotypes, mainly those including the ARH, AHQ and ARR alleles. This was expected as these alleles are associated with resistance to classical scrapie, and some may also be rare in the UK national flock (12, 23). Neither the ARQ/VRQ nor the ARR/VRQ sources used in this study gave rise to 1-4-7-scrapie profile supporting preexisting data regarding ARQ/VRQ sources (6, 8). These observations suggest that the presence of at least one valine at position 136 is sufficient to produce the alternative random profiles commonly associated with classical scrapie. This study was performed in the context of the recommendations of the OIE guidelines for the characterization of BSE infection. However, strain identification using a panel of inbred mice is mandatory to fully characterize the agent. Such studies are currently ongoing in our laboratory, and the results will be published as soon as they are available.

Experimental transmission of BSE in sheep in a limited number of animals via the oral route, which is considered to be the most likely route of natural infection, suggests that the ARQ/ARQ genotype is associated with susceptibility to BSE (2, 23). However, it has been shown recently that VRQ/VRQ sheep can also succumb to the disease albeit with prolonged incubation period compared with ARQ/ARQ animals (3). Therefore, our finding that the 1-4-7-scrapie profile is associated with the ARQ/ARQ genotype is very significant as, to date, all ovine TSE cases that share some similarities with BSE derive from ARQ/ARQ animals (15, 16, 25). The final resolution of these cases depends on bioassay in transgenic and wild-type RIII mice.

The disease incubation period for 1-4-7-scrapie isolates was significantly longer than that of ovine, but not bovine BSE isolates, while the mean incubation period of the bovine BSE isolates used in this study was significantly longer than that of ovine BSE. It should be noted that primary transmissions of BSE to RIII mice were selected randomly for comparative purposes in this study. As such, a comprehensive analysis of the incubation periods resulting from primary transmission of bovine vs. ovine BSE to RIII mice has not been performed here. However, it may be noteworthy that bovine BSE isolates were derived from pooled bovine brains, while ovine BSE isolates were derived from individual ovine sources. We cannot comment at this time on the influence of pooled sources vs. individual animal-derived inocula on the subsequent incubation period on primary isolation in mice. Notably, all ovine BSE isolates were also derived from ARQ/ARQ ovine sources so the comparison of 1-4-7-scrapie to ovine BSE is most suitable and indicates a significant difference in incubation period between the two groups. Incubation periods on primary isolation are particularly dependent on the prion titer in the isolate, and, as such, their power as a discriminatory parameter is more questionable for primary isolation data. However, in conjunction with the histopathological and immunohistochemical differences described, relative incubation periods may provide additional evidence for the distinction between 1-4-7-scrapie and ovine BSE. Furthermore, results presented in this study indicate that ARQ/ARQ ovine sources analyzed gave rise to significantly shorter incubation periods in mice than VRQ/VRQ sources.

The relatively low attack rates of scrapie observed in this study are compatible with those in published data (8). This seems to be a feature inherent to scrapie and may reflect the highly adapted nature of the agent to the host species. In contrast, BSE readily transmits to RIII mice with higher attack rates than scrapie provided that the titer of the agent is high. Lower titer may inevitably lead to decreased attack rates, and, in such cases, lesion profiles are unreliable. However, as we showed in this study, detailed pathology and IHC could discriminate BSE from scrapie at the level of the individual mouse. This discrimination is possible even in preclinically affected mice, particularly using IHC, provided that there are enough distinguishing markers present. These data are not the subject of this work, as lesion profiles require the inclusion of at least five clinically positive mice to be reliable.

Differential Western immunoblotting is a technique widely used to distinguish scrapie in sheep from natural cases of cattle BSE and experimental BSE in sheep with the differences in PrP^Sc^ molecular profiles appearing consistent and distinct. In contrast, more variation of these parameters, such as a lower molecular mass or variable reactivity to the N-terminal antibody, is seen in some sheep TSE isolates. One such anomalous scrapie strain is CH1641, an isolate derived from a British sheep that displays Western blotting profile similarities to experimentally transmitted BSE in sheep, although the high ratio of di-glycosylated PrP^Sc^ is not seen (24). However, the IHC results for the six samples giving unusual molecular profiles were indicative of scrapie and not a CH1641 isolate. Current research has shown that further discrimination is possible between ovine BSE and CH1641-like scrapie, using Western immunoblotting. This is achieved by the use of antibodies targeting different epitope sequences of the abnormal protein (1). While none of the scrapie cases in this study fit the full range of CH1641 criteria, the use of such new developments will aid future investigation and differentiation of BSE from other unusual variants of scrapie in sheep.

It has previously been suggested that the cause of BSE may have been an unidentified strain of scrapie. However, we do not believe that the similarity between the 1-4-7-scrapie and BSE profile indicates that BSE has emerged from 1-4-7-scrapie. This belief is based on our experience of analyzing H&E and IHC data. The H&E analysis (and the photos presented in this work) shows that 1-4-7-scrapie is clearly different from BSE in specific neuroanatomic areas, which are either not included in the profiles (ie, ventral midbrain) or are part of a larger scoring area and, as a result, are contributing only partially to the score of that region (ie, the cochlear nuclei are only one of the nuclei included in area G1). This may reflect the fact that the lesion profiles have been developed several decades ago by using C57BL mice to discriminate mouse-adapted scrapie strains and not to discriminate BSE from scrapie on primary isolation. However, this system has been applied historically (18), and, given that at the time the 1-4-7-scrapie profile was not identified, it was believed that the lesion profiles arising from BSE were unlike any known scrapie profiles (8). We believe, therefore, that the similarities of the two profiles do not reflect biologic similarities but, instead, are coincidental, and, if different areas were selected, the profiles could be distinctly different. This notion is reinforced by the completely different IHC patterns presented by the two agents.

This study is the first to describe a consistent lesion profile from classical scrapie isolates on primary isolation in RIII mice and to identify a profile that shares peaks at gray-matter areas 1, 4 and 7 with the BSE profile. In addition this 1-4-7-scrapie profile was associated with the ARQ/ARQ sheep PrP genotype. We show that further analysis of lesion profiles and pathology derived from these isolates distinguishes them from BSE. This study proposes that the distinction between BSE and classical scrapie on primary isolation using RIII mice based on lesion profile analysis is not as straightforward as previously thought. It is highly beneficial to consider other parameters, primarily vacuolation in areas not represented in the lesion profile, IHC and presence/absence of plaques in conjunction with profile analysis.
